# A Preliminary Evaluation on the Antifungal Efficacy of VT-1161 against Persister *Candida albicans* Cells in Vulvovaginal Candidiasis

**DOI:** 10.3390/biomedicines12020389

**Published:** 2024-02-07

**Authors:** Marica Sinoca, Angela Maione, Edvige Gambino, Marianna Imparato, Marilena Galdiero, Elisabetta de Alteriis, Emilia Galdiero, Marco Guida

**Affiliations:** 1 Department of Biology, University of Naples Federico II, Via Cinthia, 80126 Naples, Italy; marica.sinoca@unina.it (M.S.); angela.maione@unina.it (A.M.); edvige.gambino@unina.it (E.G.); marianna.imparato@unina.it (M.I.); elisabetta.dealteriis@unina.it (E.d.A.); marco.guida@unina.it (M.G.); 2 Department of Experimental Medicine, University of Campania “Luigi Vanvitelli”, 81100 Naples, Italy; marilena.galdiero@unicampania.it; 3 Center for Studies on Bioinspired Agro-Environmental Technology (BAT Center), 80055 Portici, Italy; 4 National Biodiversity Future Center (NBFC), 90133 Palermo, Italy

**Keywords:** *Candida albicans*, recurrent vaginal candidiasis, persister cells, biofilms, antifungals, HaCaT model

## Abstract

Persister cells are a small fraction of the microbial population that survive lethal concentrations of antimicrobial agents. *Candida albicans* causes vaginal candidiasis, including recurrent vulvovaginal candidiasis, and may survive common antifungal treatments. The triazole VT-1161 is an antifungal agent that specifically targets fungal CYP51, as opposed to the human CYP enzyme. This work illustrates a new role of VT-1161 in eradicating the biofilm created from the persister cells of a primary biofilm of a clinical vaginal isolate of *C. albicans*. Antifungal activity was determined by the minimum inhibitory concentration (MIC), and the primary biofilm was treated with amphotericin B to obtain persister cells that were able to form a new biofilm. Results obtained using the new azole VT-1161 showed that VT-1161 not only eradicated a secondary biofilm formed from the persister-derived biofilm and counteracted the adhesion of *C. albicans* in vitro to human cells but also ameliorated *C. albicans*-induced infection in vivo in *Galleria mellonella* larvae, suggesting that it could be proposed as an alternative therapeutic strategy for the treatment of recurrent candidiasis.

## 1. Introduction

More than 50% of cases of candidemia are caused by *Candida albicans*, including vulvovaginal candidiasis (VVC) [1]. In particular, it was observed that *C. albicans*, compared to non-*albicans* species, adheres better to the vaginal mucosa [2].

According to some research, about 70% of women will have at least one VVC episode in their lifetime [3]. The most common risk factors for vaginal candidiasis are frequent use of antibiotics, pregnancy, diabetes mellitus, oral contraceptives, and inadequate treatment. The continuous use of antibiotics promotes both the appearance of VVC as a first event and the reappearance of the disease (recurrent vulvovaginal candidiasis, RVVC) [2]. Nowadays, RVVC is very common, affecting 138 million women worldwide [4,5].

In most cases, fluconazole is used to treat RVVC, but recent studies have shown increased resistance to this antifungal in women with this disease [6].

The ineffectiveness of common antifungal drugs used for the treatment of RVVC is related to different survival mechanisms, including the ability of *Candida* to form biofilms on both biotic and abiotic surfaces, such as medical devices [7,8]. Some studies, performed on murine models, report the involvement of biofilms in the pathogenesis of VVC and RVVC [9].

Indeed, the formation of biofilm reduces the penetration of antifungals, and therefore promotes increased resistance to conventional drugs [10]. Other factors contributing to the failure of therapy are overexpression of efflux pumps and the presence of persistent cells in fungal biofilms [11,12].

Persistent cells are a phenotypic variant of wild-type cells not in active growth, capable of surviving at high concentrations of antimicrobials [13]. In clinical settings, persistent infections are usually associated with recurrence of infections and the development of chronic infections [11,14,15].

When the microbial population is treated with high concentrations of an antimicrobial compound, a biphasic pattern of survival can be observed, as most cells die, while only a very small percentage remain alive, this representing the subpopulation of persistent cells [16]. In addition, if persistent cells are isolated, regenerated and repeatedly treated with high drug concentrations, the same response to the drug as the original population will be observed [17].

Recent studies have found that adherence to a support by *Candida* is crucial for the formation of persistent cells, though they have not been detected in planktonic cultures, but only associated with biofilms [18]. Further, it has been observed that these cells are formed during the adhesion phase and that their number is constant during the development of the biofilm [19].

Considering the role of biofilms in the pathogenesis and recurrence of VVC and the low rates of healing using current FDA-approved therapies, new therapeutic antibiofilm agents are needed [7]. In this context, Galdiero et al. (2020) have proposed the possible synergistic action of an antimicrobial peptide (gH625) in combination with common antifungal drugs for the eradication of a *C. albicans* biofilm, derived from persister cells, which had survived to a previous conventional treatment with amphotericin B [17].

A similar approach was followed also in the case of the persister-derived biofilm of another *Candida* species, *C. auris*, which was successfully treated with *Lavandula angustifolia* essential oil [20].

Tetrazole oteseconazole (VT-1161) represents a new generation of fungal CYP51 inhibitors with high specificity for fungal CYP51 compared to human CYP enzymes [21,22].

VT-1161 was shown to exhibit potent in vitro activity against *C. albicans* in initial tests and against fluconazole-resistant *C. albicans* from acute and recurrent vulvovaginal candidiasis [23]. VT-1161 has proved to be clinically effective and safe not only in a phase 2 study in women with recurrent vulvovaginal candidiasis (RVVC) but also in a phase 3 study on the treatment of culture-verified vulvovaginal candidiasis (VVC) episodes, thus leading to its approval by the FDA in April 2022 (clinicaltrials.gov identifier NCT03840616, NCT03562156, NCT03561701) [24].

In our previous work, we tested the efficacy of VT-1161 as an antibiofilm agent, showing its ability to inhibit and eradicate mono- and dual-species *Candida* biofilms [24].

In this work, we aimed to extend the potential of such a compound by assessing its ability to eradicate a secondary biofilm generated by persistent cells occurring in a biofilm of a *C. albicans* vaginal fluconazole-resistant isolate. In addition, VT-1161 was investigated in the displacement of the adhered *C. albicans* isolate, both planktonic and biofilm-derived, from non-tumorigenic human keratinocyte (HaCaT) cells.

Further, to assess the prophylactic and therapeutic efficacy of VT-1161, infection in *Galleria mellonella* was used as an in vivo model.

## 2. Materials and Methods

### 2.1. Strains and Culture Conditions

A clinical *Candida* isolate resistant to fluconazole and previously confirmed by phenotypic and molecular methods to be *C. albicans* [25] was used in this study. It was stored as frozen stocks at −80 °C and was maintained on rose bengal agar (RB, Sigma-Aldrich, St. Louis, MO, USA). Colonies of *C. albicans* were taken and inoculated into liquid tryptic soy broth (TSB, VWR chemicals, Leuven, Belgium) supplemented with 1% *w*/*v* glucose. The culture was incubated at 37 °C, 200 rpm overnight. Cells were harvested by centrifugation at 5000 rpm, 4 °C for 10 min, followed by washing in phosphate-buffered saline (PBS, Oxoid Ltd., Basingstoke, UK) three times. For biofilm formation, the final suspension was adjusted to 1 × 10^6^ CFU mL^−1^ in RPMI 1640 without L-glutamine (Capricorn Scientific GmbH, Ebsdorfergrund, Germany); 100 μL of such suspension was loaded into a polystyrene 96-well microplate and incubated at 37 °C for 24 h to allow biofilm formation.

To determine viable *Candida* cells, proper serial dilution in PBS and plating on YPDA (1% yeast extract (Sigma Aldrich, Co., St. Louis, MO, USA), 2% Bacto Peptone (Gibco, Life Technologies Corporation, Detroit, MI, USA), 2% glucose (SERVA Electrophoresis GmbH, Heidelberg, Germany), and 2% agar (Condalab, Madrid, Spain) were performed. Agar plates were incubated at 37 °C for 48 h, and the number of viable cells expressed as colony-forming units (CFU).

### 2.2. Cell Culture

A non-tumorigenic human keratinocyte cell line (HaCaT cells) was used in this study.

The cell line was cultivated in Dulbecco’s modified Eagle’s medium (DMEM, Sigma Aldrich, Co., St. Louis, MO, USA), supplemented with 10% fetal bovine serum (Sigma Aldrich, Co., St. Louis, MO, USA), 1% L-glutamine (Sigma Aldrich, Co., St. Louis, MO, USA), and 1% penicillin–streptomycin (Sigma Aldrich) in a humidified incubator at 37 °C and 5% CO_2_. After 70% confluence, the cells were enzymatically detached with a 0.25% trypsin–EDTA solution (Sigma-Aldrich, Co., St. Louis, MO, USA) and cultured into new flasks. The culture medium was replaced twice a week. The cell line was monitored daily using an inverted microscope (Meiji Techno Microscopes, San Jose, CA, USA).

### 2.3. Minimum Inhibitory Concentration (MIC)

Amphotericin B (Amph B, Thermo Fisher Scientific, Waltham, MA, USA) stock solution at a concentration of 4 mg mL^−1^ and VT-1161 (Target Moltebu-bio, Chemicals Inc., Mont Belvieu, TX, USA) at a final concentration of 100 μg mL^−1^ were prepared in DMSO 5% *v*/*v*.

The MIC of Amph B and VT-1161 was determined in accordance with a broth microdilution protocol from the Clinical and Laboratory Standards Institute (CLSI M27-A4) [26]. Briefly, concentrations of Amph B from 0.025 µg mL^−1^ to 4 µg mL^−1^ and of VT-1161 from 0.001 to 4 μg mL^−1^ were added to wells of a 96-well microplate containing 1 × 10^6^ CFU mL^−1^ of *Candida* cells. The plate was incubated at 37 °C for 24 h and growth was determined at 590 nm wavelength with a microplate reader (SYNERGYH4 BioTek, Winooski, VT, USA).

The MIC_90_ of the two compounds was determined as the lowest drug concentration that produced 90% inhibition of growth relative to control.

### 2.4. Detection of Persister Cells

Detection of persister cells in *C. albicans* clinical isolate biofilm was performed as previously described [17], with some modifications. Briefly, the 24 h *C. albicans* biofilm (B1), grown as previously described, was challenged with Amph B buffered to pH 7 with 0.165 M morpholinopropanesulfonic acid (MOPS, Sigma-Aldrich Co., St. Louis, MO, USA) at concentrations ranging from 5 to 200 µg mL^−1^ for 24 h at 37 °C in RPMI medium (Capricorn Scientific GmbH, Ebsdorfergrund, Germany).

Biofilm was thoroughly washed with PBS, then disrupted by scraping and vortexed vigorously for 30 s, and the quantification of persisters was determined by the number of surviving cells expressed as log CFU per well.

### 2.5. Development of Persister-Derived Biofilm of C. albicans

The secondary biofilm (B2) was formed from the small number of cells (persisters) that survived the Amph B treatment in B1 and was allowed to develop in situ in the 96-well microplate. Therefore, to obtain B2, the wells were profusely washed with PBS, and 100 µL of YPD (2% yeast extract (Sigma Aldrich, Co., St. Louis, MO, USA), 4% Bacto Peptone (Gibco, Life Technologies Corporation, Detroit, MI, USA), and 4% glucose (SERVA Electrophoresis GmbH, Heidelberg, Germany)) medium were added to each well every 24 h, prolonging the incubation at 37 °C for 10 days. The recovery of the B2 was estimated every 48 h by determining the viable *Candida* cells by CFU assay.

### 2.6. Eradication of the Persister-Derived Biofilm with the Tetrazole VT-1161

The eradication activity of VT-1161 was evaluated by exposing B2 for 24 h to different concentrations of VT-1161 0.5, 1.0, and 2.0 µg mL^−1^ and the residual biomass quantified by crystal violet assay, as previously described [24]. The percentage of eradication was calculated as biofilm reduction % = (OD_570_ control − OD_570_ sample/OD_570_ control) × 100, where OD_570_ control and OD_570_ sample corresponded to the untreated and treated biofilm, respectively.

### 2.7. Displacement Assay

To investigate the ability of VT-1161 to displace adhered *Candida* cells from HaCaT cells, we first evaluated the adhesion ability of both planktonic and B2-derived cells.

Both suspensions were prepared in DMEM supplemented with 10% fetal bovine serum, 1% L-glutamine, and 1% penicillin–streptomycin.

The adhesion ability was assessed as follows. HaCaT cells were seeded in a 24-well (2.5 × 10^5^ cells in each well) and incubated at 37 °C in a 5% CO_2_ atmosphere overnight. The next day, cells were washed with PBS, and a final suspension of 1.25 × 10^6^ CFU mL^−1^ *Candida* cells was added to each well. After 2 h of incubation at 37 °C and 5% CO_2_, wells were thoroughly washed with PBS, and the adhered *Candida* cells were recovered using 0.25% trypsin–EDTA solution. After proper serial dilution, suspensions were plated onto YPDA for *Candida* CFU assay.

For the displacement test, after 2 h adhesion of both *Candida* cells to HaCaT, the microplate wells were washed, and VT-1161 at final concentrations of at 0.5, 1.0, and 2.0 μgmL^−1^ was added to the wells, which were further incubated for 1.5 h. Then, the residual adhered *Candida* cells were recovered and determined by CFU assay.

### 2.8. Galleria mellonella Survival Assay

To evaluate the VT-1161 antifungal effect in vivo, *G. mellonella* survival assays were performed. First assessed was VT-1161 toxicity at concentrations ranging from 0.5 to 2.0 µg mL^−1^. Twenty larvae in each group, with a body weight between 200 and 300 mg, were injected directly into the hemocoel proleg region using a 50 µL Hamilton syringe with a 26 g needle. Larval killing assays were carried out incubating larvae at 37 °C, and death was monitored daily over 3 days by visual inspection of the color and lack of movement after stimulation. The infection was performed with *Candida* cells recovered by B2, inoculating 10^6^ yeast cells/larva. VT-1161 at a concentration of 1 µg mL^−1^ was administered to larvae 2 h before infection (prophylactic treatment) or 2 h after infection (therapeutic treatment). Groups of (i) untreated larvae, to evaluate general viability, (ii) larvae injected with PBS buffer only, and (iii) larvae injected with VT-1161 only, to check the compound toxicity, were included in the experiments. The experiments were performed in triplicate.

### 2.9. Statistical Analyses

All data were analyzed using GraphPad Prism Software (version 8.02 for Windows, GraphPad Software, La Jolla, CA, USA, www.graphpad.com, accessed on 2 January 2024). The results were derived from three independent experiments, and they are shown as average ± standard deviation (SD). One or two-way ANOVA following Dunnett’s or Tukey’s test was used for multi-comparison tests. Asterisks show significant differences, (* = *p* < 0.05, ** = *p* < 0.01, *** = *p* < 0.001, **** = *p* < 0.0001). Survival curves were plotted using the Kaplan–Meier method and log-rank (Mantel–Cox) test for comparation between the groups.

## 3. Results

### 3.1. Determination of Minimum Inhibitory Concentration (MIC)

As shown in Table 1, the MIC of the vaginal *C. albicans* isolate was 2 μg mL^−1^ for both Amph B and VT-1161. Our analysis in a previous study [23] demonstrated that this *C. albicans* vaginal isolate had a strong biofilm production ability in vitro.

### 3.2. Detection of Persister Cells in C. albicans Biofilms

**Figure 1:**
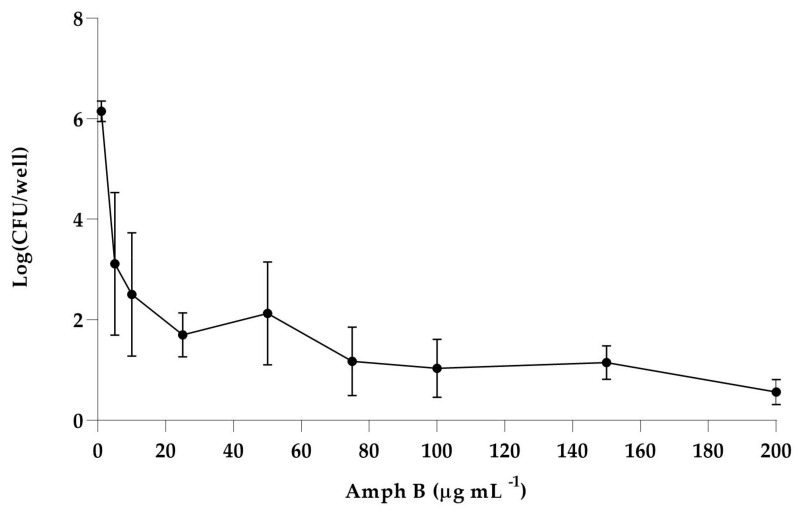
Detection of persisters in *Candida albicans* biofilm. Survival of *Candida albicans* cells in biofilm following treatment with Amph B at different concentrations. Results show the mean of three independent experiments; error bars represent SD.

Amph B was used to challenge such primary biofilm formed on a polystyrene surface. Indeed, Amph B is one of the most effective antifungal agents, and can kill fungal cells in all phases of growth, so representing the optimal choice also to isolate persisters from a clinical isolate biofilm. Therefore, as shown in Figure 1, the primary biofilm of *C. albicans* established on the polystyrene surface was challenged with increasing concentrations of Amph B up to 100 × MIC, and for each concentration, the viable residual *Candida* cells in the biofilm were determined (Figure 1).

The biphasic-killing curve revealed the occurrence of a subpopulation of cells in the biofilm not susceptible to the antifungal challenge, the so-called persisters.

The persister percentage was approximately 0.001% of the total population of mature biofilms, in agreement with previous studies [18,27]. Our results further confirm that the *C. albicans* vaginal isolate persister cells were present in biofilms, representing phenotypic tolerant variants. According to a study by LaFleur et al. [28], where strains with a survival rate of >6% were defined as high persisters and those showing survival < 6% were classified as low persisters, the vaginal isolate can be considered a low persister.

### 3.3. Development of a New Biofilm from Persisters (B2) and Antifungal Activity of VT-1161 on B2

**Figure 2:**
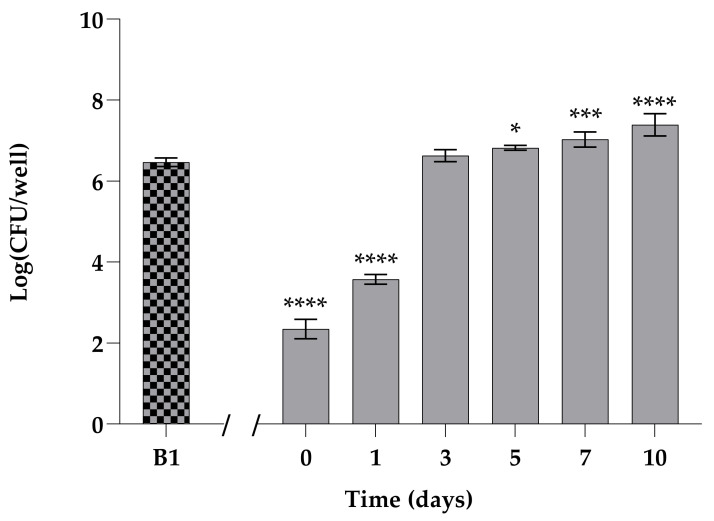
Development of a new biofilm from persisters. Viable cells increase during formation of the biofilm generating from cells of the primary biofilm survived at Amph B treatment at time = 0. Results show the mean of three independent experiments; error bars represent SD (* = *p* < 0.05, *** = *p* < 0.001 **** = *p* < 0.0001, Dunnett’s test).

A secondary persister-derived biofilm (B2) was obtained by dispensing fresh nutrient medium into the wells of a microplate where the primary biofilm (B1) of *C. albicans* had been challenged with 200 µg mL^−1^ Amph B (Figure 2). In such conditions, a new biofilm (secondary biofilm) progressively developed, starting from persisters. We observed that the number of living cells in the secondary biofilm increased over time, achieving the same value as that of the primary biofilm already after 3 d (Figure 2).

**Figure 3:**
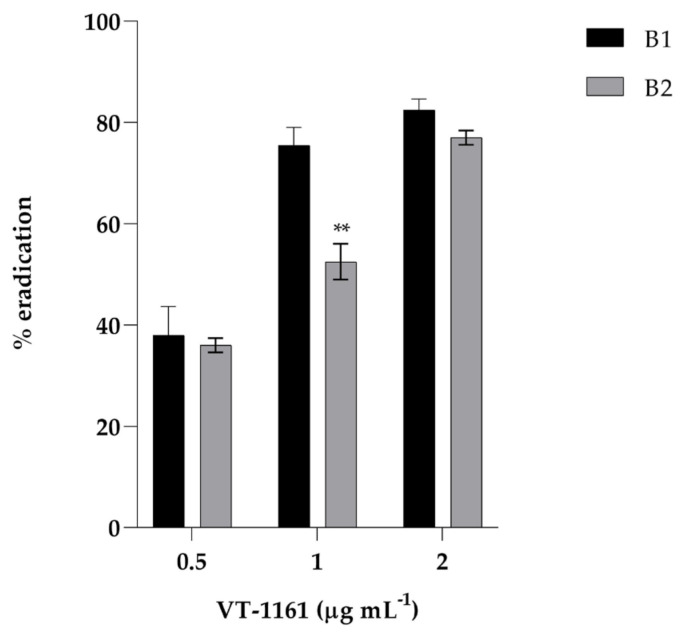
Biofilm eradication activity of VT-1161 against *Candida albicans* primary (B1) and secondary (B2) biofilm. Results show the mean of three independent experiments; error bars represent SD (** = *p* < 0.01, Tukey’s test).

In Figure 3, the results of the effect of the drug on the eradication of the persister-derived biofilm (B2) of the clinical strain compared to the primary biofilm (B1) are reported.

A dose-dependent effect of the drug on the eradication of both biofilms was observed. It is clearly evident that at sub-MIC concentrations of 1 µg mL^−1^, the eradication of B1 was about 70%, while the eradication of B2 was about 55%, showing a significant difference in VT11-61-removing ability. On the contrary, when we utilized the compound at MIC concentration, its eradication ability was similar for both biofilms. However, already at the low concentration of 0.5 µg mL^−1^, VT-1161 was able to exert its eradication property at almost 40% for both biofilms.

### 3.4. Adhesion of Candida albicans Planktonic Cells and B2-Derived Cells to HaCaT Cells and Anti-Adhesion Effect of VT-1161

**Figure 4:**
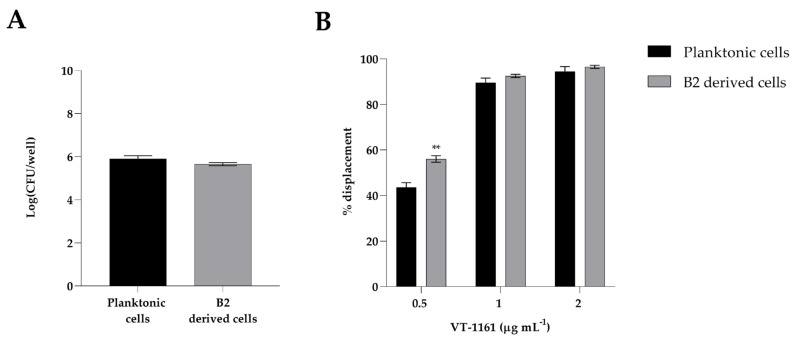
Adhesion of *Candida albicans* (planktonic cells and B2-derived cells) to HaCaT cells (**A**). Assessment of anti-adhesion effect of VT-1161 of *Candida albicans* planktonic and B2-derived cells on HaCaT. Infection of HaCaT cells with the strain of *Candida albicans* without treatment was used as a positive control (**B**). Results show the mean of three independent experiments; error bars represent SD (** = *p* < 0.01, Tukey’s test).

*C. albicans* (planktonic cells and B2-derived cells) was able to adhere to HaCaT cells with a higher adhesion capacity in both conditions, as depicted in Figure 4A.

Our results showed (Figure 4B) that VT-1161 could be used for the displacement of *Candida* cells from HaCaT. The same concentrations of the compound already used in the biofilm eradication assay were very effective in displacing yeast cells.

While at a concentration of 0.5 µg mL^−1^, the adhesion was partially reduced with respect to the control samples, at both concentrations of 1 and 2 µg mL^−1^ the displacement reached almost a 90% effect, although without reaching statistical significance between planktonic and biofilm-derived *Candida* cells. Altogether, these data suggest that VT-1161’s potential for treating *C. albicans* interaction with epithelial cells and for B2-derived yeast cells.

### 3.5. In Vivo Activity of VT-1161

**Figure 5:**
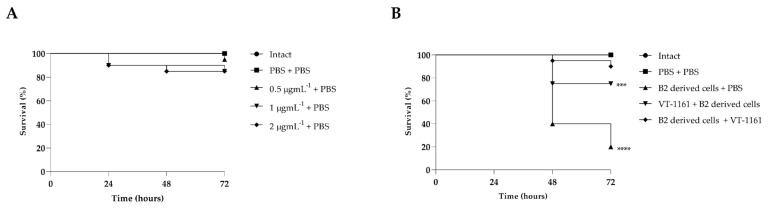
Toxicity of VT-1161 on *G. mellonella* larvae (**A**). Kaplan–Meier plots of survival curves of *G. mellonella* larvae infected with biofilm-derived *C. albicans* (1 × 10^6^ CFU/larva). Treatments consisted of intact larvae, PBS, *C. albicans* (biofilm-derived) + PBS, VT-1161 before or after *Candida* infection (**B**). Asterisks represents significant difference vs. larvae injected with *C. albicans* (*** = *p* < 0.001, **** = *p* < 0.0001, log-rank Mantel–Cox test).

The survival assay in *G. mellonella* was used to assess not only the safety but also the ability of VT-1161 to prevent and combat the infection of *C. albicans* B2-derived cells in vivo. Results are reported in Figure 5A,B.

First, it was clearly shown that VT-1161 was non-toxic for larvae, since survival of larvae injected with the compound alone at all three concentrations tested remained about 80% after 72 h (Figure 5A). Already after 72 h, as reported in our previous research [25], larvae showed a reduced survival of about 80% in this condition of infection (1 × 10^5^ CFU per larva). This result was confirmed here also for B2-derived cells (Figure 5B).

Further, in Figure 5B), it is shown that VT-1161 administration clearly hindered fungal infection. Indeed, a very significant increase in larva survival up to 70% was observed already after 72 h, in both cases when the compound was given before or after infection.

## 4. Discussion

Up to 75% of women have a VVC infection at least once in their lifetime, commonly due (85–95%) to the pathogen *Candida albicans*, and approximately 9% of women with VVC can have recurrent VVC (RVVC), defined as three episodes of VVC in 12 months. Despite progress in fungal drug treatments for these recurrent diseases, frequent failure in antifungal therapy is linked to the presence of populations of drug-tolerant and persister cells [29]. The Infectious Diseases Society of America guidelines recommend that RVVC must be managed with therapy with a topical agent or with oral fluconazole for a long period. The long-term use of fluconazole can cause liver toxicity, drug interactions and danger during pregnancy and also carries the risk of development of resistance and formation of persister cells.

Some studies outline the link between the occurrence of persister cells of various microorganisms and recurrence of the disease, together with the immune response, the antibiotic use, environmental change, and stress such that patients cannot completely eliminate the pathogen [30].

Persisters are commonly associated with recalcitrant infections often related to biofilm formation, in which a small population of persister cells can resist lethal antimicrobial treatment and be responsible for unfavorable prognosis, because the persisters can produce a new biofilm with a similar persister level and an equivalent antifungal susceptibility. *Candida* persister cells exhibit a nonhereditary, multidrug tolerance to antifungals and are dormant with a quiescent metabolism. The mechanism of *C. albicans* persister formation is today unknown, and the low levels of persisters and their transience make them difficult to isolate [31].

VT-1161 has demonstrated potent in vitro activity against most isolates of *Candida* species associated with RVVC. In particular, it is indicated to reduce the incidence of RVVC [32] due to its strong duration of efficacy and broad spectrum of antifungal activity, including against fluconazole-resistant strains.

In this study, we first show that persister cells are present in a biofilm grown from a *C. albicans* vaginal clinical isolate detected by treatment with increasing concentrations of Amph B. Our research showed that a low persister cell population (0.001%) was present after the complete removal of the isolate using Amph B 200 µg mL^−1^, even if their presence was not observed in all *C. albicans* isolates, as reported by other researchers.

Then, we followed the development of a new biofilm derived from persisters in the microwell plate model, showing how persisters can progressively develop a new biofilm in situ, mimicking the recolonization of a surface, which may be responsible for recurrent infections. In our previous study [24], we demonstrated that the new azole VT-1161 was able to inhibit and eradicate mono- and polymicrobial biofilms made of fungal/bacterial cells often found in recurrent infections, and here we confirmed the strong ability of VT-1161 to eradicate a secondary biofilm.

As reported by other studies, persister cells recovered from B1 required a longer period of time to reach a normal level of metabolic activity to form and develop a secondary biofilm, maybe due to a cellular stress imbalance [33], and could not be eradicated by conventional antifungal drugs at very high concentrations, making them responsible for the recalcitrance of infections to antifungal treatment [12].

Wu et al. hypothesized in another study a connection between biofilm growth by *C. albicans* in the vaginal epithelium and the formation of persister cells, supporting a role of *Candida* biofilm formation in the recurrence of vaginal candidiasis [34].

The current study established that VT-1161 is able to eradicate a secondary biofilm well derived from persisters present in the primary one compared to the primary biofilm from the *Candida* clinical isolate analyzed.

*C. albicans* infections are initiated by adhesion of the fungus to the epithelium followed by invasion of these host cells, so we tested if cells from B2 of our clinical isolate were able to adhere to HaCaT, confirming the results of a great adherence capacity.

To evaluate whether the effect of VT-1161 was also effective on mammalian cells, we used the same concentrations of the eradication test in vitro.

VT-1161 showed a great efficacy in removing adhered *Candida* cells to HaCaT cells in vitro, confirming its functionality in treatment of the infections.

The in vivo safety properties of VT-1161 and its ability in preventing or treating infection were further evaluated in an invertebrate host model. *G. mellonella* health was monitored every 24 h until 72 h postinjection. No dosages tested showed toxicity, and VT-1161 confirmed in vivo the great capacity to interfere with *C. albicans* infection caused by cells from secondary biofilm.

In closing, our current work on VT-1161 and a *C. albicans* vaginal isolate reveals an appreciated function of this drug in the control of fungal virulence, representing a potential novel option to treat recurrent episodes of RVVC against clinical *Candida albicans* isolates, including fluconazole-resistant strains.

## Figures and Tables

**Table 1:** Minimum inhibitory concentration (MIC) ± standard deviation (SD) of Amph B and VT-1161 against *C. albicans* clinical isolate.

	MIC (μg mL^−1^)	
*C. albicans* clinical isolate	Amph B	VT-1161
	2.0 ± 0.3	2.0 ± 0.2

## Data Availability

Data are contained within the article.
